# Cause and Being of Teeth

**Published:** 1895-11

**Authors:** Henry H. Burchard

**Affiliations:** Philadelphia


### CAUSE AND BEING OF TEETH.[1]

BY HENRY H. BURCHARD, M.D., D.D.S., PHILADELPHIA.

The subject of this essay is that of the cause and being of teeth, and, incidentally, some few observations as to their embryology and histology, some few loose comments upon the interdependence of function and structure. It is most certain that in the struggle of organisms for existence there is no such surplusage of vital force that any of it will be wasted, so that in its distribution, as evidenced in the formation of tissues and organs, we shall surely find that whatever organ or part is formed has its use, and comes only into being to supply an animal need. Instances of seeming variation from this, such as the colors of flowers or birds, and so on, which appear to be but for mere ornament, are delusive, for even in such cases utility is the end, not mere adornment.

There is distributed to and expended upon a developing part, and afterwards in its maintenance, no more energy than is needed. Nature's economy is finely adjusted, and there is rarely built more than is necessary. Variations, if they occur, take the line of minus more frequently than plus. Many writers upon physiography speak of the lavishness, the wastefulness of Nature, as evinced in

---

[1] Read before the Edwin T. Darby Society, University of Pennsylvania, February 25, 1895.

the loss of energy by the diffusion of the sun's heat, but it is more than probable that our egotism is at fault here, and, although this planet may not be the beneficiary of all the sun's energy, it is not wasted for general cosmic purposes. To return to an organism, just so much energy is expended in its formation as is needed to put it in harmony with its future environment. With this in mind, whatever is our specialty, we may examine the parts immediately concerning us with the assurance that every part, even those most microscopic, forms a means to an end, and no inquiry as to the matter can be too exhaustive.

The human tooth is our subject, or, for that matter, the teeth of any of the vertebrata. To make analogies closer, teeth of one type are selected, those having a continuous enamel surface, supported by dentine, and this in its turn by cementum and the membrane which gave origin to it. These and their supports, down to the mandible, are a means to an end, and this end the preparation of the food for the chemical solution which we call digestion. As teeth are the basis of our reasoning here, we assume the conditions which demand teeth. For these latter, obviously the first consideration is a substance and surface composed and shaped so that the usual food of man may be divided and crushed,—comminuted. That this office demands the greatest extent of hardness of the substance performing it premises that Nature will set aside special provision for its production, and that this provision will show an elaborateness according with the extent of organization.

Contrary to usual methods of exposition, our argument carries us now, not to the part immediately contiguous, but to the general support, the maxillæ and the motive apparatus. These must needs be of sufficient resistance and power to withstand the forces applied in mastication. Added to this, the lower jaw is a lever of the third class, and the resistive power must be increased just in the ratio of the distance of the weight from the fulcrum and of the power from the fulcrum,—the power, the force of the muscles in mastication; the fulcrum, the temporo-maxillary articulation; the weight, the resistance offered by the food in mastication,—so that the longer the inferior maxilla, the more dense it should be; also the more powerful the muscular action, the more dense and thicker should be the bone. Direct muscular force plays no part in mastication, as all the teeth are anterior to the lines of insertion of the muscles of mastication. The differentiation of an organ for the formation of enamel and the beginning of a bony formation in the substance of the embryonic jaw occur before there are any evidences of cells being differentiated for the production of dentine. That, in spots, what Dr. Sudduth has described as interstitial formation of bone is taking place about as early as the purpose of the epithelial infolding becomes evident.

This arrangement is a verification of the reasoning which makes dentine formation secondary to that of the enamel and the maxilla. The recalling of the existence of the slide was an accident which, fortunately, supports the arrangement selected.[1] The amount of force expressed in the leverage demands for resistance more than a mere vitreous surface, such as the enamel,—this should have a firm basis of support,—hence dentine, or some substance resistive and firm, yet of less density than the enamel, becomes a necessity. As the office of this is for support, not crushing, Nature's economy does not permit of any more elaboration than enough to meet the needs. This structure requires support and of another grade, less firmness. You will see there is a progression of the degrees of elasticity provided, and the modified bone cementum is supplied. These combined structures need fixation and support, and the alveoli are furnished. There is no instance in the body where provision is not made for the prevention of shocks, as you will see from an examination of Nature's provisions in the arrangement of articulation, their shapes and angles, histological anatomy, etc., in the viscera, of their means of support by properly arranged ligaments, and further by elastic capsules.

So, in common with other organs, this has its ligament and elastic support combined in the pericementum, a nutritive membrane, an elastic semi-capsule. The arrangement of some of the fibres of this membrane is of such a character as to form a cushion for the tooth, and this is its provision against shock. The cancellated structure beyond the alveoli acts as a subsidiary to the cushion, as may be seen by its peculiar arrangement.

The force of impact is next upon the bone, and beyond this to the base of the brain, but there are several provisions between, which reduce the amount of shock at that point. The angle of the jaw, breaking the direct line of the force, is one; the elastic cartilages second; and the angle which the perpendicular line of the ramus of the mandible makes with the plane of the base of the cranium another. An instance of where this force comes at a right angle is in what is called the knock-out blow of the pugilist. Delivered as this blow is, it is as though a straight bar extended from

---

[1] American System of Dentistry, vol. i., Fig. 356.

the centre of the articulating head of the maxilla to the point where the blow is received, about beneath the bicuspid teeth.

The elasticity of the tissues supporting teeth subserves another purpose, and that is an adjuvant to the local circulation. As you no doubt know, there are several auxiliaries to the cardiac force in the circulation. Prominent among these are the mechanical forces exerted by elastic tissues, muscles and their sheaths. Any force which produces changes in shape, position, or size of a part serves the same end. The force of mastication, stretching the fibres of the pericementum, pumps the circulating fluids of the parts.

You will perceive from this that an increasing density of teeth and their supporting structures, through a lessening of this elasticity, will reduce the amount of this auxiliary force to the vascular system. This may proceed to such an extent that a condition of vulnerability is produced. A lessening of evident cellular elements and an increase of highly organized tissue may be carried so far that a tooth becomes almost like a foreign body in its own socket. This may, in fact, be the determining element in locating a specific inflammation in the pericementum.

You will perceive that in the normal contact of the lateral aspects of the teeth the contact is by but a small surface or point, so that each tooth has independent movement in mastication. Irregularities of the teeth frequently lessen the independent movement by offering an increased surface of contact. Apropos of this checking of movement, if you will consider the nature of the relations established through the use of fixed bridges, you will see that there results inevitably a change of nutritive relations as to the pier teeth and their supports. This may or may not be of much moment in practice, but still it forms an item for consideration.

The transmutations of epithelium form one of the most remarkable series of phenomena in all biology. A mere cursory (students will readily detect a twofold significance of this word in connection with embryology), even a superficial examination will show the multitude of offices performed by epithelium. Up to the part played by this structure in the formation of the central nervous system, and we may trace the steps of organic evolution very, very low in the scale of cellular organisms,—that is, a stage above the unicellular,—the periphery is the receptive part, and so on through various elaboration of function there is a modification of what was originally a peripheral part. Among all the peculiar variations of epithelial structures there will be found none more remarkable than that producing the enamel of the teeth. As the first office of the tooth is that of dividing and crushing, we look naturally for the earliest formation to be that of a structure designed for its production. An examination of a fœtal jaw will show this to be true, that, antedating any other evidence of cellular differentiation pointing to tooth-formation, there will be found a definite, linear, bounded activity of the epithelium covering the summit of the jaw.

That this *is* a defined hyperactivity is seen by the rapidly multiplying cells causing a rising ridge. However, this rising is a secondary effect, with no other significance than the presence of a greatly increased number of germinal cells underlying a given area.

The upheaval is increased in extent according to the depth or cubical contents of the involution. The next stage in the progress of this differentiation, the base of the cylindrical (in fact laminal) infolding appears to meet the resistance of a cone, as a depression begins at the middle of the base. The supports of the masticating and incisive organs, approaching the latter in importance of function, are developed almost simultaneously, the mesoblastic tissue partakes of the activity and increases, so the epithelial cap, as it now is, is left deep in the cellular mass. At the same time indications of the future jaw are beginning to appear. From a common band elements corresponding to individual teeth are separated, and single teeth are now in process of development. The enamel organ assumes more of the form of a tooth crown. The space between the two layers of epithelium, made by reduplication, becomes distended. A fibro-vascular coat forms about its exterior. The periphery of the mound it encloses is seen to have a distinctive layer of cells formed upon it, blood-vessels become more evident in the mound, and the dentinal and enamel organs are ready for their work of elaboration. The alveolar walls are seen in process of formation, and soon indications of a periosteum appear.

In connection with the future of the processes under discussion, there are several controverted matters. It is possible that some of you will supply the missing links. Theoretically, a new generation of practitioners should begin where the last closed their labors. It does not take many years to make a professional generation, and you have a good basis in the work which has already been done, and so the expectation is no extravagant one. Prominent among the matters *sub judice* is that of the exact manner of formation of the enamel and dentine. The details involved and to be solved are those connected with the peculiarity of cell action, which determines the forms of the enamel and dentinal elements. The transition there is between matter on one side of cells and formed products on the other. This intermediate ground is the matter which should engage your attention. The solution will no doubt be found through some improvement in the technique of histology.

As to enamel formation there are two prominent hypotheses. One that the enamel is deposited in the extremities of ameloblasts, that these cells elongate as the deposition proceeds, so that, according to this view, an enamel prism, throughout its length, is a petrified epithelial cell. The next hypothesis is that enamel is a secretion exuding from the distal ends of the ameloblasts, and that this secretion becomes hardened, petrified into enamel. Sudduth states that individuals of calco-globulin spherites coalesce when in contact.

Personally, I incline to a belief which includes these two processes,—that the ameloblasts, which form a layer of persistent, prismatic epithelial cells, are in a state of constant reproductive activity, and that the progeny holding calco-globulin as cell contents are being placed at the distal ends of the ameloblasts. It appears to me that this is more in accordance with the general rule of epithelial development.

Another matter requiring solution is that of the formation of the transverse processes in dentine. You are, of course, familiar with the minute structure of dentine. In this connection there are several obscure matters connected with the study of the minute anatomy of the dentine, its embryology, and, last, its vulnerability, which you gentlemen may at some time solve.

Of the period of dentification, covered by the beginning and ending of root formation, there is a paucity of literature and a lack of observation. This offers an inviting field for any of you who are interested in histology.

Another matter which seems to be undetermined, and one regarding which many have speculated, is the composition of Nasmyth's membrane. You will observe that a tooth-sac has, between the formed enamel and the tissues immediately contiguous to the outer layer of the sac, the following parts: a fibro-vascular membrane, which is said to form the pericementum; an external epithelial coat to the enamel organ; the remnants of the stellate reticulum, and the ameloblastic layer itself. Now, unless it can be shown that one or more of these layers are lost during the process of eruption, each must form a layer of Nasmyth's membrane, and with this addition, that the layer which becomes the pericementum may deposit a layer of cementum. I believe this has been observed. Thus the skin of the teeth may consist of five layers. Even should the fibro-vascular layer be pierced, and the tooth-crown emerge through the aperture, it would have the three epithelial layers at least as coating. We have discussed something of structure, and now to the matter of the forms of the teeth. The classes of teeth will be found to have forms and positions adapted to special purposes: incisors for primary division, cutting, or incising; bicuspids for half cutting, half crushing; molars for little cutting and much crushing.

In this immediate connection I ask your careful attention and study to the work of Dr. Bonwill as to occlusion. I am debarred on philosophical grounds from agreement with Bonwill as to the cause of teeth, but in relation to most of his views as to their being there can be no conflict and no question of the immense value of his work and studies in human articulation. Virtually, his argument is that design begets want; as you no doubt know, the general opinion of evolutionists is that want begets design; and the truth of it—well, perhaps it lies between.

As to these three classes of teeth, examine the cuts of Bonwill as to overbite, and see how the amount of this corresponds with the function of the particular tooth; also, the arrangement of the cusps which gives the greatest amount of utility commensurate with structure.

One point Dr. Bonwill demonstrated to the writer, which does not appear to be in works on odontography,—as to the function of the cuspids. As you are aware, these teeth are assigned a part in prehension,—that of locking. Dr. Bonwill shows that an important function is that of guiding the bite,—that is, in the carnivora, where these teeth are most prominent (as teeth), the surfaces of the canines meet, and are guided to closure in such a manner as to cause the molars to act as shears. Dr. Kirk has the skull of a baboon in which the same arrangement is seen. In this case the provision is doubtless for cracking nuts, not bones, as in the carnivora. This function pertains in some degree, or rather the necessary degree, to the cuspids of man. The palatal aspect of the superior canine, it will be seen, is composed of two inclined planes,—one mesial, the other distal. Against these planes occlude parts of the labial aspects of the inferior cuspid and bicuspid. The action of this occlusion would be upon an interposed substance in the main incisive, but added to this would be a squeezing, as in a rolling-mill. This gives the first bruising to the morsel of food. When the apex of the superior cuspid is in contact with its interior occluders some space will be seen to exist between molars and bicuspids, perhaps enough to admit the bruised morsel of food. Now, these occluding surfaces guide the cusps of the molars and bicuspids into a shear-edge occlusion.

You will observe that the amount of overbite of the first bicuspid is greater than that of the second; necessarily, it will receive a greater strain than the second during mastication. Nature's provision against this strain as to support will be found in a bifurcation of the root of the first bicuspid, thus widening and increasing the base of resistance.

This is but one fact as to the roots, and I think a new one. Apropos of this, Dr. Kirk suggests to me the reason for superior molars having three roots. That the mandible and its masticating surfaces is the movable stone of a mill, the superior maxilla and its masticating surfaces the base stone, which requires more firmness than the movable; hence the extra root.

The root arrangement is in such manner as to offer the proper support to each type, and each is so shaped as to offer the proper resistance to the applied strains. Those of the superior incisors, somewhat triangular on section, the base anterior. That of the cuspids nearer an ellipse, for the strain is divided. In connection with this root note the prominence of the process overlying it and the length of the root. It is the tooth of the mouth which has least support from other teeth during one stage of mastication.

The inferior incisors, to most effectually resist the strains against their roots, should show sections triangular at the neck and a reversed triangle at the apex. The arrangement is a compromise, as these roots have an elliptical section.

Dr. Bonwill has explained, or no doubt will explain to you at some time, the mutual support derived from the arch arrangement of these inferior incisors.

There is a mechanics of embryology which determines in large part the shapes, sizes, positions, etc., of anatomical structures, or at least helps to determine them.

This is a lengthy subject, and one of which but little has been written.[1] Its treatment and discussion will involve much labor and thought, and will serve for some future discourse.

---

[1] Dr. Ryder, in the Proceedings of the Academy of Natural Sciences, contributed some twelve or fourteen years ago an exhaustive study upon the mechanics of tooth-formation. Later, the work was epitomized by Dr. C. N. Peirce.

The conditions described in the paper are as to present existence, and the effects of mechanical causes are included in the results. It does not require extensive observation to demonstrate that the mechanical aspect plays a prominent part in the determination of form. The practical result, the object of all this work which concerns us, is, of course, the preservation of the teeth.

In this connection permit me to call your attention to one fact which seems to be frequently overlooked, and that is, this preservation should be with a view to utility; this the comminution of the food; and all our efforts at conservation should be directed with that end in view. Teeth retained should do work,—should be put in such condition that they may do this effectually. This involves the filling of teeth, so that the filling should represent a restoration of lost parts, for if we diverge from this the tooth has its usefulness lessened to the extent of divergence.

As before stated, Nature does not build uselessly, so the amount of energy she has expended in elaboration of the masticatory apparatus is just so much as its importance demands. That in the mammalia she stops tooth formation anterior to the fauces is positive evidence that the work of mechanical subdivision of the food should be done in the space occupied by teeth. As she has substituted in the stomach chemical for mechanical operation is proof that she designs that food shall be presented to this organ in condition for chemical solution. This makes a curious medley of material, disconnected, and perhaps not as clear as it might be. Running through it, I think you will see that Nature shows no better examples of the conservation of energy than in organized creation. The evidences are somewhere, in fact everywhere, and it will serve as a pleasurable exercise for you to detect them.

Do not think that these matters are of no moment; they are as important as any with which we have to deal. Oliver Wendell Holmes, in one of his books, describes an experiment in the developing of infusoria. At its conclusion, the experimenter, as he watches the tubes, exclaims or muses that the fate of the Roman Catholic Church is contained in that vial. The experiment was one in the investigation of the possibility of spontaneous generation. And so it may be in the scientific aspect of dentistry. There may be some one point apparently obscure and of little importance, and yet, intrinsically, it may outweigh in importance the results of years of practice of the most skilled clinician.

All things are comparative, and human vanity is constantly receiving shocks as to its estimation of comparative worth. And now, as the clergyman says, one word more and I have done. Just one hint. A text-book will state that O is a bivalent element; but few words, gentlemen, but change the fact of that condition and the universe would change with it.

[The subject matter of this essay was illustrated by numerous black-board diagrams and drawings, most of them modified outline drawings from the several text-books of anatomy and histology; others by appropriate slides of histological preparations.]

